# Generation of Embryonic Stem Cells and Mice for Duchenne Research

**DOI:** 10.1371/currents.md.cbf1d33001de80923ce674302cad7925

**Published:** 2013-09-10

**Authors:** Marcel Veltrop, Jos van der Kaa, Jill Claassens, Laura van Vliet, Sjef Verbeek, Annemieke Aartsma-Rus

**Affiliations:** Department of Human Genetics, Leiden University Medical Center, Leiden, Netherlands; Department of Human Genetics, Leiden University Medical Center, Leiden, Netherlands; Department of Human Genetics, Leiden University Medical Center, Leiden, Netherlands; Department of Human Genetics, Leiden University Medical Center, Leiden, Netherlands; Department of Human Genetics, Leiden University Medical Center, Leiden, Netherlands; Department of Human Genetics, Leiden University Medical Center, Leiden, Netherlands

## Abstract

Duchenne muscular dystrophy (DMD) is a muscle-wasting disease in which muscle is continuously damaged, resulting in loss of muscle tissue and function. Antisense-mediated exon skipping is a promising therapeutic approach for DMD. This method uses sequence specific antisense oligonucleotides (AONs) to reframe disrupted dystrophin transcripts. As AONs function in a sequence specific manner, human specific AONs cannot be tested in the mdx mouse, which carries a mutation in the murine Dmd gene. We have previously generated a mouse model carrying the complete human DMD gene (hDMD mouse) integrated in the mouse genome to overcome this problem. However, as this is not a disease model, it cannot be used to study the effect of AON treatment on protein level and muscle function. Therefore, our long term goal is to generate deletions in the human DMD gene in a mouse carrying the hDMD gene in an mdx background. Towards this aim, we generated a male ES cell line carrying the hDMD gene while having the mdx point mutation. Inheritance of the hDMD gene by the ES cell was confirmed both on DNA and mRNA level. Quality control of the ES cells revealed that the pluripotency marker genes Oct-4 and Nanog are well expressed and that 85% of cells have 40 chromosomes. Germ line competence of this cell line has been confirmed, and 2 mice strains were derived from this cell line and crossed back on a C57BL6 background: hDMD/mdx and mdx(BL6).

## Introduction

Duchenne muscular dystrophy (DMD) is a muscle-wasting disease in which muscle is continuously damaged due to the lack of functional dystrophin 1 ^,^ 2. Since the regenerative machinery cannot fully cope, patients suffer from continuous muscle damage, eventually leading to replacement of muscle by fibrotic and adipose tissues 2 ^-^ 4.

Research groups are exploring various therapies to slow down disease progression. Therapies are mainly based on restoration of dystrophin expression and on improving muscle quality. Antisense-mediated exon skipping, aiming to convert a severe DMD phenotype into a milder Becker muscular dystrophy phenotype is currently closest to clinical application 5. In DMD patients the reading-frame of the dystrophin mRNA is disrupted resulting in a prematurely truncated, non functional dystrophin protein. Utilizing antisense oligonucleotides (AONs), the splicing of an out of frame exon during maturation of the pre-mRNA is prevented to restore the reading frame, allowing the production of an internally deleted but partially functional dystrophin protein.

AONs target exons in a sequence specific manner and therefore human specific AONs cannot be tested in mouse models 6. Consequently, the *mdx *mouse, which carries a mutation in exon 23 of the murine *Dmd* gene and is the most widely used model for DMD research, cannot be used as a preclinical model to test human specific AONs. Therefore we have developed the hDMD mouse 7, which carries the complete human *DMD* (hDMD) gene integrated in the mouse genome (B6.DBA2.129-hDMD^tg/tg^, from now on referred to as hDMD mouse). When crossed with *mdx* mice, the human dystrophin can compensate for the lack of mouse dystrophin and prevent pathology 7.

A drawback of the hDMD mouse is that it is not a disease model, due to its expression of human dystrophin. This makes it impossible to study the restoration of human dystrophin and muscle function and quality upon AON treatment. A mouse model to allow in vivo assessment of human specific AONs would be very valuable. Preferably this model would not express mouse dystrophin and would have a deletion in the human *DMD* gene (ΔhDMD) in the mutation hotspot region (between exon 45 and 53) 8 ^,^ 9, in contrast to the *mdx* mouse, which has a point mutation in exon 23 in the mouse *Dmd* gene.

We could have used the available hDMD embryonic stem (ES) cell line to generate a deletion in the* hDMD* gene, which has an Ola129 background. To obtain mice lacking both human and mouse dystrophin protein, these mice would have to be crossed with the *mdx* mouse, which have a C57BL10 background 10. Thus, the final ΔhDMD/*mdx* mice would have a mixed OLA129/C57BL10 background. This scheme would have to be followed for each new hDMD mutant, resulting in differences of backgrounds for different mutants, which would make the comparison between the different mutant models and other DMD and control mouse strains difficult. We therefore decided to generate an hDMD/*mdx* ES cell line as a founder cell line to generate control hDMD/*mdx* mice on an C57BL6 background mutations in the *hDMD* gene.

Although derivation of mouse ES cells has already been described in the early eighties 11 ^,^ 12 the procedure was for a long time not very efficient and mainly done with Ola129 mice. In 2006 Bryja et al 13 described a simple method by which ES cell lines could be derived from different transgenic mouse strains. The factor that improved the chance of success most was the use of knockout serum replacement instead of fetal calf serum in the culture medium. In 2008 Ying et al 14 reported a new breakthrough. They used inhibitors of the GSK-3β pathway and the ERK pathway and showed that by using these inhibitors, ES cells could easily be derived and even cultured without Leukemia Inhibitory Factor (LIF). Here we describe the generation of an hDMD/*mdx* ES cell line and resulting hDMD/*mdx* and *mdx*(BL6) mouse strains using a protocol based on these published protocols.

## Material and methods

**Mice**

C57BL6 mice were obtained from Charles River (Belgium), whereas both the hDMD mice and C57BL/10ScSn-DMD*mdx*/J *mdx* were from a colony housed at the LUMC animal facility. Mice were housed under standard conditions and were fed regular chow. All animal handling was approved by the animal ethical committee of the LUMC. Effort was put in minimizing the amount of distress caused to the animals as much as possible.

**Generation of the hDMD/*mdx* ES cell line**

Blastocysts were isolated from time mated hDMD male mice and super ovulated mdx female mice. Super ovulation of the *mdx* mice as well as isolation of the blastocysts was done according to a procedure described by Nagy et al 15. Briefly, female mice of 5-6 weeks of age were intraperitoneally (i.p.) injected with 0.1 ml folligonan (5 IU/100 μl, Intervet, The Netherlands) and 48 hrs later with 0.1 ml of chorulon (5 IU/100 μl, Intervet). Directly after the second injection the female mdx mice were housed with a male hDMD mouse during the night. In the morning females were checked for a vaginal plug and were separated from the male mouse. Two and half day later female mdx mice were sacrificed and the ovaries were isolated and flushed with phosphate buffered saline (PBS) to collect the blastocysts.

These blastocysts were cultured to generate ES cell lines according to a method described by Bryja et al 13. Blastocysts were layered on murine embryonic fibroblast (MEF) feeder cells in a well of a 24-well plate, in knockout DMEM supplemented with 2 mM L-glutamine, 1 mM sodium pyruvate, non-essential amino acids, 50 units/ml of penicillin as well as streptomycin, 1000 units/ml of LIF) and 15% knockout serum replacement (all from Life Technologies Ltd, Bleiswijk, The Netherlands). Usually after 6 days blastocysts had hatched and a small colony of cells had formed. These were trypsin digested and cells were placed in a new MEF coated well of a 24-well plate. To stop trypsin activity cells were cultured overnight in ES medium supplemented with 10% fetal bovine serum (FBS Gold, Life Technologies Ltd). The next morning medium was replaced by ES medium with knock serum replacement. These steps were repeated several times till sufficient ES cells were available for freezing down and analysis.

**Analysis of gender and presence of hDMD gene and mdx mutation**

ES cells were harvested and cultured for 45 minutes in a gelatin coated tissue culture dish to remove MEF. Floating cells were collected and chromosomal DNA was isolated and dissolved in 50 µl of H_2_O, 1-2 µl was used for the PCR reactions specified below. All enzymes, their buffers as well as the dNTPs were supplied by Roche (Almere, The Netherlands).

To determining whether the ES cells had inherited the *hDMD* gene, a PCR was carried using primers specific for exon 44 and 45 of the* hDMD* gene. Sample mix consisted of dNTP, Phire taq and the provided buffer and forward and reverse primers. The primer set of exon 44 consisted of the forward primer 5’gcgatttgacagatctgttg3’ and the reverse primer 5’ctcaacagatctgtcaaatcg3’ while that of exon 45 consisted of the forward 5’aatctgcggtggcaggagg3’ and reverse primer 5’tctgtctgacagctgtttgc3’. The PCR program was as follows: 30 seconds at 98°C, 30 cycles of 5 seconds at 98°C, 5 seconds at 60°C and 10 seconds at 72°C followed by 1 minute at 72°C.

Inheritance of the *mdx* mutation in exon 23 of the *Dmd* gene was determined by PCR and a melting curve analysis (MCA). The sample mix consisted of Fast-start Taq, the provided PCR buffer, dNTPs, DNA intercalating dye LG-Green (Bioke, Leiden, The Netherlands) and the forward primer 5’aaagttctttgaaagagcaa3’ and reverse primer 5’cagatagttgaagccatttt3’. Per reaction 8 µl of the PCR mix was mixed with 2µl DNA and 15 µl of mineral oil. The PCR program was as follows: 10 minutes at 95°C, 40 cycles of 20 seconds at 95°C, 30 seconds at 60°C and 40 seconds at 72°C followed by 5 minutes at 72°C and 1 minute at 95°C. After the PCR reaction the products were analyzed using Lightscanner and Call-IT 1.5 software of Idaho Technology inc.(Salt Lake City, USA) to assess whether the *mdx* mutation was present. All samples were tested in duplo and with each reaction 2 wild type, 2 heterozygous *mdx* and 2 homozygous* mdx* DNA samples were included as controls.

To determine the gender of the ES cell line a PCR for the male specific Sry gene 16 was carried out. The sample mix consisted of Taq the provided PCR buffer, dNTPs, and the forward primer 5’ctgtactccaaaaaccagcaaag3’ and reverse primer 5’agtaagtaggtaagctgctggtcgt3’. The PCR program was as follows: 5 minutes at 94°C, 24 cycles of 40 seconds at 94°C, 1 minute at 59°C and 80 seconds at 72°C followed by 7 minutes 72°C and 10 minutes 22°C.

Expression of the *hDMD* gene on RNA level was determined by culturing the ES cells for 1 week in so called spin embryoid bodies (EB) in MEF medium. These EBs contain cells of all three germ layers including muscle. Of these EBs RNA was isolated by Tripure (Roche) and cDNA prepared as described 17. The dystrophin mRNA was determined by nested PCR as described by Spitali et al 17 using primers described by Aartsma-Rus et al 18.

**Assessment of expression of the pluripotency markers Oct-4 and Nanog by FACS analysis**

ES cells were harvested by trypsin treatment and cell numbers were determined using a counting chamber. Cells were diluted to 2*10^5^ cells per sample. All subsequent steps were carried out on ice and in between each step cells were washed 3 times with ice-cold PBS supplemented with 2% FCS (wash buffer).

Cells were paraformaldehyde fixed (Sigma-Aldrich, Zwijndrecht, The Netherlands) and subsequently permeabilized and a-specific binding places blocked by incubation with PBS supplemented with Triton X-100 (0.05% v/v, Sigma), 5% normal goat serum and 5% FCS for 30 minutes. Next, cells were incubated for 1 hour in 10 μl of 100 times diluted anti-Oct-4 of SantaCruz (Bio-Connect BV, Huissen, The Netherlands) and anti-Nanog of eBioscience (Vienna, Austria). The Oct-4 stained cells were incubated with the secondary antibody goat-anti-mouse whereas the Nanog stained cells were incubated with goat-anti-rat antibody. Both secondary antibodies (Life Technologies Ltd) were Alexafluor 488 labelled and 1000 times diluted in wash buffer. After the last wash cells were collected in 200 μl wash buffer and analysed using a LSRII FACS machine and FACSdiva software (both of BD Biosciences, Breda, The Netherlands). Cells stained by the secondary antibody only were used as control.

**Chromosome spreads and karyotype**

ES cells were cultured for 1 hour in ES medium supplemented with 0.1 μg/ml colcemid (Life Technologies Ltd) to arrest the cells in the metaphase. Cells were washed with PBS and harvested by trypsin treatment. Both culture medium and the PBS used to wash the cells were collected and trypsin treated cells were collected in this mixture. After centrifugation, cells were suspended in pre-warmed 0.075 M KCl under constant shaking and incubated for 10 minutes at 37ºC. Subsequently, a few drops of freshly prepared methanol/acetic acid (3:1) were added and cells were collected by centrifugation. Cells were then suspended in 1 ml of the methanol/acetic acid mix and incubated for 20 minutes at room temperature to fixate the cells. Fixation was completed by refreshing the methanol/acetic acid two times, upon which cells were pelleted and suspended in 700 µl of methanol/acetic acid. From a height of at least 50 cm a drop of the cell suspension was dropped on an ether cleaned glass slide and air dried. Next a glass coverslip was mounted on the spread utilizing Vectashield mounting solution (Brunschwig Chemie, Amsterdam, The Netherlands). The cells were analyzed under fluorescent microscopy (Leica, Rijswijk, The Netherlands) and at least 20 metaphases were analyzed of which 80% should have 40 chromosomes.

**Assessment of expression of dystrophin protein by the hDMD/*mdx* ES cell line by Western blot**

To determine whether our hDMD/*mdx* mice express human dystrophin protein one male and one female mouse were sacrificed and skeletal muscles (gastrocnemius, tibialis anterior and triceps), diaphragm, heart and liver (non-muscle control) were isolated. Protein lysates were generated using the Next Advance Bullet Blender (Bio-Connect, Huissen The Netherlands) in combination with zirconium beads of 1.4 mm in diameter (OPS Diagnostics, Lebanon, NJ, USA) in lysation buffer (containing 20 % SDS in 0.1 M Tris-HCl, pH 6.8) and protein yield was determined using the Pierce BCA kit (Thermo Fisher, Etten-Leur, The Netherlands). Protein lysates isolated from *mdx* gastrocnemius and human healthy muscle (kind gift of L. Hoogendijk Department of Clinical Genetics, LUMC, Leiden, The Netherlands) were used as respectively negative and positive controls.

Protein samples were resolved on a Criterion XT 3-8% Tris-Acetate gel in XT Tricine Running buffer, using the Criterion Cell Western blot system (all from BioRad Laboratories, Veenendaal, The Netherlands) as described by Hulsker et al (submitted manuscript). Briefly, after running, samples were blotted to Nitrocellulose paper using the Trans-blot Turbo Transfer System (Biorad). Human dystrophin protein was detected using Mandys106 antibody at a 1:50 dilution (MDA monoclonal antibody resource, Prof. Glenn Morris) the Novocastra antibody NCL-Dys1 (dilution 1:125, Leica, Rijswijk, The Netherlands) was used to detect both mouse and human dystrophin protein. As a loading control α-actinin was detected using the AB72592 antibody (dilution 1:7500) of Abcam (Cambridge, England). Protein bands were visualized using the Odyssey (Westburg, Leusden, The Netherlands) after staining with the IRDye 800CW labeled secondary goat anti-mouse antibody for dystrophin and IRDye 680 TL labeled secondary donkey anti-rabbit antibody for α-actinin (both from Westburg, both diluted 1:5000).

## Results

**Validation of the ES cell derivation protocol of Bryja**

Prior to generating the hDMD/*mdx* ES cell lines we tested the ES generation protocol described by Bryja 13 for hDMD mice. For this, blastocysts were isolated from hDMD females after a timed mating with male hDMD mice. We obtained 18 blastocysts, of which 10 were cultured according to the protocol of Bryja. The remaining eight were cultured according to the same protocol but the medium was supplemented with the 2i components. Using the regular Bryja protocol we were able to derive 9 (90% yield) ES cell lines and with addition of 2i we obtained 5 (75% yield) ES cell lines. However the ES cell colonies obtained with 2i, had a nicer morphology than those cultured without 2i (Figure 1) and we thus selected the 2i supplemented medium for use in subsequent experiments.

**Morphology of hDMD ES cell lines with and without 2i in the culture medium:**
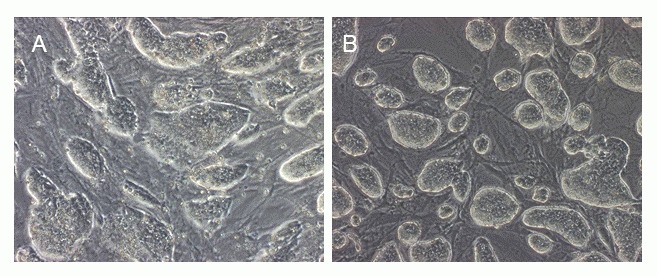
Representative clones cultured without (A) and with 2i components (B). The colonies cultured in the presence of 2i had sharper boundaries and were more compact then those cultured without 2i.

**Generation of the hDMD/*mdx* ES cell line**

To obtain our desired hDMD/*mdx* ES cell line, mdx female mice have to be super-ovulated prior to mating with hDMD male mice. In the transgenic animal facility of the LUMC super ovulation of C57BL6 mice is normally initiated at the age of 5 weeks. However, in 5 week old *mdx* mice (C57BL10 background) super ovulation was hard to induce, as all 5 mice that received hormones and were found with a vaginal plug, were negative for blastocysts.

It has been reported by Malusky et al 19 that *mdx* mice of 6-8 weeks of age gave a lower yield of oocytes compared to mice of 3-5 weeks of age, even using twice the amount of hormones regularly used. However, the oocytes of older mice were more reliable for blastocyst formation. Therefore, we repeated the experiment with 7 week old* mdx* mice using twice the hormone dose. From 5 mice we now obtained 6 blastocysts and 4 morulas. These were seeded individually in wells of a 24-well plate as described. Since the yield was so low and effects of 2i on the resulting cell lines was at time of performing the experiments still under debate, we decided to culture without 2i. Using these conditions five of 6 blastocysts and 1 of the 4 morula yielded an ES cell line.

**Characterization of the ES cell lines:**
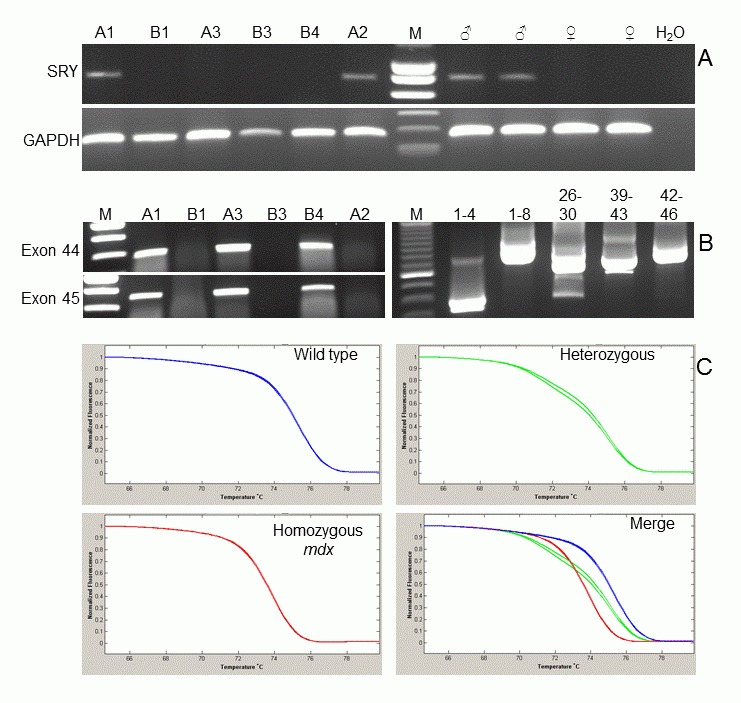
DNA of the ES cell lines was extracted and used to determine the gender of the ES cell lines (A), inheritance of the hDMD (B) and mdx mutation (C). A GAPDH PCR was used as a DNA quality control. To determine the gender the male specific SRY PCR was performed, revealing that clone A1 and A2 are of male gender (A). DNA of male and female WT mice was used as positive and negative controls. Inheritance of the hDMD gene (B) was determined by PCR with primers specific for exon 44 and 45 of the hDMD gene. Data reveal that clone A1, A3 and B4 carry the hDMD gene in their genome. Furthermore, clone 1 expresses the hDMD gene on RNA level (B, right panel). The mdx specific point mutation in exon 23 was detected using PCR primers over the mutation site. PCR was performed in the presence of a DNA intercalating dye followed by melting curve analysis (C). As a control, DNA of a wild type mouse, heterozygous mdx mouse and a homozygous mdx mouse were taken along. The data reveal that male clone A1 carries the mdx mutation and that the female clones are heterozygous.

**Characterization of the ES cell lines**

As we intent to use the derived ES cell lines to mutate the *hDMD* transgene to generate new DMD mouse models, a male gender is preferred for the cell lines. To determine the gender of the ES cell lines, DNA of the MEF depleted ES cell lines was extracted and analyzed with the gender specific SRY PCR. Data revealed that 2 clones (A1 and A2) were of male gender (Figure 2A).

We then confirmed whether the new derived ES cell lines contained the* hDMD* gene by determining the presence of exon 44 and 45 using human specific primers. Three of the clones were found to have the *hDMD* gene in their genome, clone A1, A3 and B4 (Figure 2B, left panel).

Next we determined whether the male clone A1 also expressed the *hDMD* gene on mRNA level. ES cells were cultured for 1 week as spin EB in MEF medium to induce differentiation of the ES cells. Of these embryoid bodies total RNA was extracted and analyzed for expression of the *hDMD* gene. We analyzed expression of the first 46 exons and clone 1 was found to express all tested exons (Figure 2B, right panel).

Finally, we confirmed the *mdx* genotype using the melting curve technique (an example is shown in Figure 2C). Data revealed that the male ES cell line A1 has the *mdx* genotype and that the female cell lines A3, B1 and B4 are heterozygous for the *mdx* genotype, while the other 2 clones are wild type.

**Quality control of the ES cell line A1**

To confirm the pluripotency of line A1, the expression of the transcription factors/pluripotency markers of ES cells, Oct4 and Nanog, was determined by FACS analysis. A1 was found to express both Oct4 and Nanog (Figure 3). Clone A1 cells were karyotyped and of the 21 nuclear spreads analyzed 18 (85.7%) had 40 chromosomes and 3 had 39 chromosomes (14.3%).

**FACS results to assess the expression of Oct4 and Nanog by the ES cell line A1:**
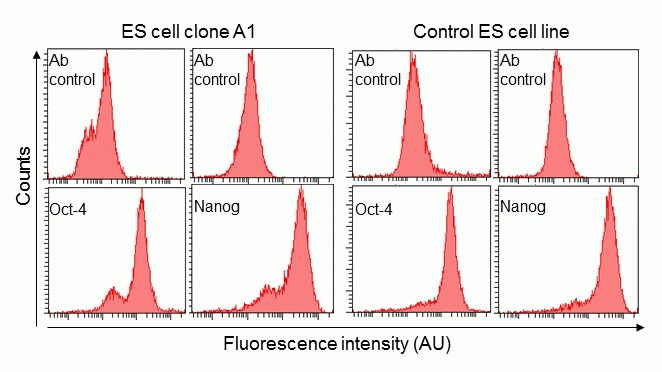
Cells of clone A1 and a control ES cell line were stained with an antibody directed against Oct4, Nanog or as a control stained with a control Antibody (Ab). Both ES cell lines express the transcription factors/pluripotency markers of ES cells Oct4 and Nanog.

**Determination of resistance of the ES cells of clone A1 to selection drugs**

In future experiments, we want to modify the *hDMD* gene by homologous recombination. This requires a targeting vector with selection markers. However, the *hDMD* gene containing ES cell line (LYac4.1) was generated using a YAC containing two resistance markers (neomycin and hygromycin). Both the LYac4.1 ES cell line and the resulting hDMD mouse therefore carry both resistance marker genes. Since our new ES cell line A1 was derived from male hDMD and female *mdx* mice the resistance markers should have been inherited.

We determined whether the A1 cells were resistant to neomycin and hygromycin. Cells were cultured in triplicate on MEF derived from neomycin, hygromycin double resistant mice. Neomycin as well as hygromycin was added in a range of 100-450 µg/ml to the culture medium and cells were analyzed 2 days and 6 days after initiation of antibiotics challenge. Two days after neomycin was administered there were some clones growing in the 100 and 200 µg/ml conditions, but these were very small. In the wells with higher concentrations of neomycin no growth was observed. At day 6 none of the wells contained ES cells of clone A1 indicating that cells have lost the neomycin resistance gene or that it is not functional. By contrast, the LYac4.1 cells survived the challenge with up to 250 µg/ml neomycin.

Presence of hygromycin for 2 days resulted in numerous colonies up to 200 µg/ml, but the cells did not show a very nice morphology. Above 300 µg/ml the MEF were starting to release blebs and cultures were hard to examine. Nevertheless A1 cells appeared to be viable up to at least 350 µg/ml hygromycin. After 6 days still numerous colonies were observed in the 100 and 150 µg/ml containing wells suggesting the hygromycin resistance gene is still present. From 200 to 300 µg/ml colony numbers had declined to around 10 per well. In all wells ES cell colonies appeared rather flat instead of the well known 3D morphology. Above 300 µg/ml no MEF or ES cells survived. The LYac4.1 cells survived hygromycin exposure up to 300 µg/ml, and had a nicer morphology compared to that of cells of clone A1. Based on these results it can be concluded that the A1 cells are hygromycin resistant but have lost neomycin resistance.

We further tested two alternative positive selection markers (blasticidin and zeocin) and a frequently used negative selection marker (ganciclovir). We tested the resistance to all three drugs using the Alamarblue fluorescence assay. Since selection after targeting takes roughly 1 week we tested cell viability after 3 and 7 days. The natural resistance was strongly influenced by the duration of the drug treatment (Figure 4). At day 3 100% killing with blasticidin was found at 6.3 μg/ml and at day 7 at 1.5 μg/ml. Zeocin had a 100% killing at 500 μg/ml and 7.8 μg/ml for day 3 and 7, respectively. In our transgenic facility negative selection is applied at 5 μM ganciclovir. At this concentration cell viability was over 80% on day 3 and after 7 days it was near 100%, indicating the cells tolerate the negative selection marker as anticipated.

**Resistance of cells of ES cell line A1 to positive and negative selection markers:**
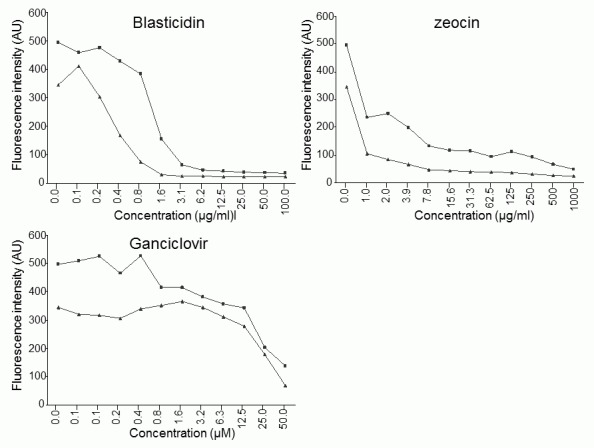
Cells were cultured in the presence of blasticidin, zeocin or ganciclovir at indicated concentrations and viability was determined at day 3 (square) and day 7 (triangle) using Alamarblue. At day 3 100% killing (minimal fluorescence) with the selection drug blasticidin was observed at 6.3 μg/ml and with zeocin at 500 μg/ml. Prolonging the selection period up to 7 days results in a lower dose killing 100% of the cells. The negative selection drug ganciclovir is well tolerated at the concentration used in our transgenic facility (5 μM) even after 7 days of treatment.

**Germ line competence of the ES cells**

The new ES cell line A1 and the future modified variants of this cell line will be used to derive new mouse models for DMD. To confirm that the ES cells are germ line competent, cells of the male hDMD/*mdx* A1 cell line were injected in C57/BL6 blastocysts and these blastocysts were subsequently transplanted in foster mothers. We obtained 24 pups, 18 males and 6 females. An example of a litter of several chimeric (patched coloration) and some wild type (black) pups is shown in figure 5. The skewing to males was anticipated due to the male ES cell line. In total 15 pups were chimeric of which 14 were male. Of the male chimeras, 11 had the hDMD gene and the *mdx* mutation, while 3 had the hDMD gene without the *mdx* mutation. The one female chimeric pup had an hDMD/*mdx*genotype. Due to the mixed background the name of the mouse using standard nomenclature is B6.DBA2.129-hDMD^tg/tg^/LUMC*B10-Dmd*^mdx^*/J. We will refer to this model as hDMD/*mdx* throughout the rest of the manuscript.

The expression of human dystrophin protein was confirmed for two hDMD/mdx mice. Using the human dystrophin specific antibody Mandys106, all hDMD/mdx muscle tested as well as the human control muscle showed expression of human protein, while no dystrophin protein was found in the wild-type mouse and mdx mouse muscle samples (Figure 6A). Staining with the NCL-Dys1 antibody, which detects both human and mouse dystrophin protein, revealed that all muscle samples, except the mdx mouse, are dystrophin positive. With both antibodies, the liver samples were negative for dystrophin protein as anticipated.

Male hDMD/*mdx* mice were crossed for 4 generations with C57BL6 females. Currently, we are intercrossing the mice to obtain homozygous hDMD/*mdx* mice. At the fourth generation on C57BL6 background there were also pups that were *mdx* but did not inherit the hDMD gene. Pups with this genotype are also intercrossed further to obtain *mdx* mice on C75BL6 background (*mdx*(BL6)) that can serve as control mice with an identical background as the hDMD/*mdx* mice.

**Expression of human dystrophin protein by hDMD/mdx mice.**
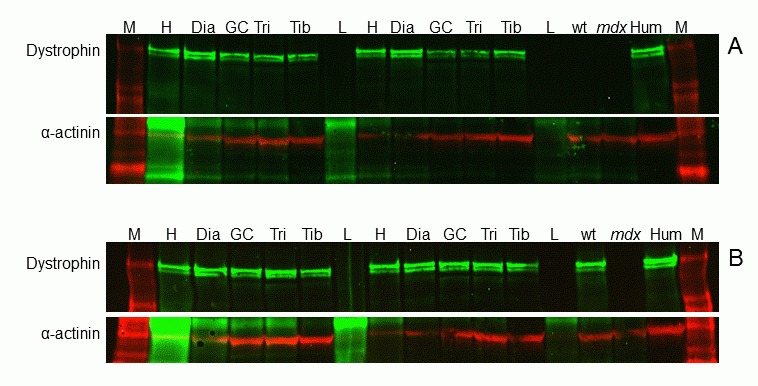
Protein was isolated from different muscles of hDMD/*mdx* mice, wild-type mice, mdx mice and human muscle. Furthermore, as a non-muscle control sample, protein was isolated from liver of hDMD/mdx mice. These protein samples were subjected to Western blotting and dystrophin protein was detected using the human dystrophin specific antibody Mandys106 (A) and the human/mouse dystrophin detecting antibody NCL-Dys1 (B). As loading control, α-actinin was detected (bottom graph of both A and B). Data reveal that using Mandys106 antibody, all muscle samples of the hDMD/*mdx* mouse and the human control sample stain positive (A), while the wild-type mouse and* mdx* mouse sample are negative. Using the NCL-Dys1 antibody all muscle samples excluding the mdx sample stain positive for dystrophin (B). Liver samples are negative for dystrophin (A, B).

## Discussion

AON-mediated exon 51 skipping of the *DMD* gene is currently tested in phase 3 clinical trials, while phase 1/2 trials are ongoing for exon 44 and about to be initiated for exon 45 and 53. Since AONs are sequence-specific, human-specific AONs cannot be tested in mouse models. Therefore, in our laboratory the hDMD mouse was generated. The transgenic* hDMD* gene gives rise to a functional dystrophin protein that can compensate for the lack of mouse dystrophin and even the lack of both mouse dystrophin and utrophin 7. Thus, the hDMD mouse is not a disease model. A model carrying mutations in the human gene in the absence of mouse dystrophin would be very valuable to allow optimization of human AONs and assessment on RNA and protein levels as well as assessment of the effects on muscle function and integrity. To this end we have generated a male ES cell line that originated from crossing the hDMD mice with *mdx* mice. This new ES cell line has been tested for its germ line capacity and yielded two new mouse strains so far: the hDMD/*mdx* mouse and the *mdx*(BL6) mouse. The hDMD/*mdx* mouse was found to correctly express the human dystrophin protein (Figure 6)*.*

In the early eighties derivation of mouse ES cells was described for the first time 11 ^,^ 12. However, the derivation was not very successful till 2006 when Bryja and colleagues 13 presented a new protocol. This method was further improved by the finding of Ying et al 14 who showed that inhibition of both the GSK-3β pathway and the ERK pathway gave an increased success of generating ES cell lines and showed that by using these inhibitors, ES cells could be cultured without LIF. Later on it was shown that these inhibitors (2i) facilitated the derivation of ES cells of rats 20 and bovine 21, which was not possible before. We have derived ES cells of the transgenic hDMD mouse (Ola129/C57BL6) using the protocol described by Bryja. Modifying the protocol by adding the 2i components to the culture medium did not positively affect the efficiency of ES cell line derivation (90% without versus 75% with 2i), but the number of blastocyst used in our study are too low (10 and 8, respectively) to draw any strong conclusions.

When generating ES cell lines from C57BL6/J mice however, we did observe a positive effect of 2i on the derivation of ES cell lines. Of 19 blastocysts cultured in the absence of 2i we obtained 10 ES cell lines (55%), while of 16 blastocysts cultured with 2i we obtained 16 ES cell lines (100%) (data not shown). So the effect of 2i on the derivation of ES cell lines could be mouse strain dependent. This finding is underscored by the finding of Kanda 22 who recently showed that deriving ES cells of Ola129 mice is not positively effected by adding 2i to the medium, whereas deriving ES cells of C57BL6 and BALBc mice is.

Notably, both the hDMD ES cell lines and the C57BL6 cell lines derived with 2i, express Oct-4 and Nanog at higher levels compared to the cell lines derived without 2i (data not shown). Moreover, the expression pattern was more homogenous among the cells cultured in 2i supplemented medium. This is in line with the data of Munoz Descalzo 23 who cultured ES cells in the presence and absence of 2i and had similar observations. They even claim that the levels may predict the pluripotency status of the cells, high levels means pluripotent, intermediate levels may indicate priming for differentiation and low or absence means differentiating cells.

At the time of generating our ES cell lines it was debated how the presence of 2i influenced the germ line capacity of the ES cells. We have injected blastocysts of C57BL6 mice with hDMD ES cells derived in the absence as well as the presence of 2i and in both cases we obtained chimeric pups. Furthermore, these pups were able to transmit the transgen to a next generation showing germ line capacity. Recently, Zhu 24 reported that routinely culturing C57BL6J as well as E14 (Ola129) ES cells in the presence of similar drugs as used in our study did not influence the germ line capacity in a negative way. Moreover, Kanda 22 reported that ES cells establishment in the presence of 2i and routinely sub-cultured in the presence of 2i still are germ line capable. So, the 2i components seem to neither influence the generation of chimeric pups, nor to have a negative influence on the germ line capacity of the ES cells.

The LUMC transgenic facility routinely collects blastocysts of C57BL6 mice starting super-ovulation when mice are 5 weeks of age. As we experienced a lot of difficulties obtaining blastocysts from the* mdx* mice (C57BL10 background), we conclude this is not an appropriate protocol to derive blastocysts from these mice. The method that we used as an alternative was based on a report of Malusky et al 19, but was far from optimal as the yield was still very low. Despite this we did obtain a proper ES clone that met our criteria e.g. being male gender, having a good karyotype of 40 chromosomes, carrying the *hDMD* gene as well as the *mdx* point mutation and generating chimeric offspring, which in turn generated germ line offspring. The ES cell line is currently used to induce mutations in the deletion hotspot of the *hDMD* gene.

The founder ES cell line of the hDMD mouse, named LYac4.1 pointing to the Yac being used to introduce the *hDMD* transgene into the mouse ES cell line, is neomycin and hygromycin resistant as a result of the introduction of that Yac. To our surprise, the hDMD/*mdx* ES cell line that was derived by mating an hDMD male mouse and an *mdx* female mouse was sensitive to neomycin treatment. Performing a neomycin gene specific PCR on DNA of the LYac4.1 ES cell line and of the hDMD/*mdx* ES cell line resulted in a PCR product of similar size. However, when the PCR products were analyzed by Sanger sequencing, it was found that there were several point mutations in the PCR product of the hDMD/*mdx* ES cell line. So the neomycin gene is not deleted from the hDMD/*mdx* genome but most likely these point mutations have resulted in loss of function.

The chimeric pups derived during the germ line test of the ES cell were bred with C57BL6 mice for 4 generations to obtain a strain more close to C57BL6 background. For each litter mice were screened for the hDMD transgene and the* mdx* point mutation. Pups of the fourth generation were divided in those having both the* hDMD* transgene and the *mdx* point mutation and those having only the *mdx* point mutation. Mice of both genotypes will be used to intercross in order to obtain mice being homozygous hDMD/*mdx* and homozygous mdx. The *mdx* mice on C57BL6 background (*mdx*(BL6)) will be used to supply the blastocysts to be injected with the ΔhDMD/*mdx* ES cells and to cross with chimeric ΔhDMD/*mdx* mice.

Since C57BL6 mice are the golden standard for many research areas, *mdx*(BL6) might be a better model than the mdx on the C57Bl10 background. Notably, when assessing the effect of the knockout genes in addition to Dmd, this is generally achieved by crossing the mdx C57BL10 with another knockout in a C57BL6 background. The offspring of mixed background is then compared to the *mdx* mice, while likely the mixing of backgrounds in and of itself also induces changes in phenotype. To avoid such a background effect in the search of a role for mutation type and aging on the arising of revertant fibers in DMD patients, Echigoya et al also generated an *mdx mouse on a C57BL6 background ^25^. *An added advantage of the C57BL6 background is that in our hands larger litters and better results with superovulation were obtained.

**Conclusion**

In summary we have derived an hDMD/*mdx* ES cell line that can be used to generate humanized DMD disease models. Furthermore, we have generated two mice strains, the hDMD/*mdx* and *mdx*(BL6) mice strains that will be useful for future studies with the humanized disease models.

## Correspondence

Marcel Veltrop, Department of Human Genetics, Leiden University Medical Center, P.O. Box 9600, 2300 RC, Leiden, The Netherlands. E-mail: m.h.a.m.veltrop@lumc.nl
